# Completeness and accuracy of data transfer of routine maternal health services data in the greater Accra region

**DOI:** 10.1186/s13104-015-1058-3

**Published:** 2015-04-01

**Authors:** Mary Amoakoh-Coleman, Gbenga A Kayode, Charles Brown-Davies, Irene Akua Agyepong, Diederick E Grobbee, Kerstin Klipstein-Grobusch, Evelyn K Ansah

**Affiliations:** Julius Global Health, Julius Center for Health Sciences and Primary Care, University Medical Centre, Utrecht, the Netherlands; School of Public Health, University of Ghana, Legon, Ghana; Ghana Health Service, Greater Accra Region, Accra, Ghana; Division of Epidemiology & Biostatistics, School of Public Health, Faculty of Health Sciences, University of the Witwatersrand, Johannesburg, South Africa; Research and Development Division, Ghana Health Service, Accra, Ghana

**Keywords:** Routine, Data, Maternal health, Completeness, Accuracy, Error rates

## Abstract

**Background:**

High quality routine health system data is essential for tracking progress towards attainment of the Millennium Development Goals 4 & 5. This study aimed to determine the completeness and accuracy of transfer of routine maternal health service data at health facility, district and regional levels of the Greater Accra Region of Ghana.

**Methods:**

A cross sectional study was conducted using secondary data comprised of routine health information data collected at facility level for the first quarter of 2012. Twelve health facilities were selected using a multistage sampling method. Data relating to antenatal care and delivery were assessed for completeness and accuracy of data transfer. Primary source data from health facility level (registers and record notebooks where health information data are initially entered) , used as the reference data, were counted, collated, and compared with aggregate data on aggregate forms compiled from these sources by health facility staff. The primary source data was also compared with data in the district health information management system (DHIMS–II), a web-based data collation and reporting system. Percentage completeness and percentage error in data transfer were estimated.

**Results:**

Data for all 5,537 antenatal registrants and 3, 466 deliveries recorded into the primary source for the first quarter of 2012 were assessed. Completeness was best for age data, followed by data on parity and hemoglobin at registration. Mean completeness of the facility level aggregate data for the data sampled, was 94.3% (95% CI = 90.6% – 98.0%) and 100.0% respectively for the aggregate form and DHIMS-II database. Mean error in data transfer was 1.0% (95% CI = 0.8% - 1.2%). Percentage error comparing aggregate form data and DHIMS-II data respectively to the primary source data ranged from 0.0% to 4.9% respectively, while percentage error comparing the DHIMS-II data to aggregate form data, was generally very low or 0.0%.

**Conclusion:**

Routine maternal health services data in the Greater Accra region, available at the district level through the DHIMS-II system is complete when compared to facility level primary source data and reliable for use.

## Background

The primary aim of health information data management is to ensure high-quality data [1,2] to support monitoring, evaluation, research and decision making. Good data management practice is a prerequisite for the achievement of high quality data [3]. A well-functioning data management system is critical in health service delivery as this ensures availability of reliable data that can be used for health service planning purposes and for evaluating the performance of the health system [4-9].

Data can be captured by paper-or electronic-based methods. Sometimes both methods are used. Commonly, in the case where both methods are used, the process starts with paper-based methods and the data is subsequently transferred electronically. During the data transfer and management processes, transcription, transmission and processing errors may occur leading to data loss, incomplete or inaccurate data. Occurrence of errors in data collection, transfer and management can be due to limited resources for data collation, lack of clarity on indicators, lack of training, inadequate manpower, poor data structure, non-adherence to or lack of standard operating procedures amongst others [10].

For prediction of the quality of data produced by either of these data capture methods, percentage error may be estimated. Errors in health service delivery data can be reduced and detected by applying double data entry, logic check (range check, detection of outliers, relational conflicts and more) and visual verification [10]. Knowledge of the processes used in the compilation of the data also provides assistance in identifying the points at which errors could occur.

Within the Ghana Health Service (GHS) routine data are produced daily at the facility level and aggregated monthly at both facility and district levels and then transmitted onwards to the regional and national levels. In pursuit of improving maternal health in Ghana to accelerate the achievement of Ghana’s targets for the Millennium Development Goals (MDGs), the focus has been on improved care to prevent unnecessary morbidity and mortality. With such a focus, it is also important that the data collected routinely for daily service provision is accurate and reliable, since it forms the basis for monitoring, evaluation and decision making towards subsequent improvement of the maternal health service provision and outcomes. Given the importance of this data the study was conducted to determine the completeness and accuracy of transfer of antenatal and delivery services data captured at facility, district and regional levels in the Greater Accra region of Ghana.

## Methods

### Study setting

The study was conducted in the Greater Accra Region (GAR), one of the ten administrative regions of Ghana. The region consisted of seventeen districts, municipalities, metropolises and sub-metropolises at the time of the study in 2012. In the region, there is a public sector teaching hospital which is not part of the Ghana Health Service (GHS). The rest of the public health sector in the region comprises a regional hospital as well as ten district and sub-metropolitan hospitals, one of which is a mission hospital. There are also four polyclinics, thirty-one health centers and thirty-eight community health and planning services (CHPs) compounds serving as primary levels of care. The region also has a thriving private sector comprising clinics, hospitals, pharmacies and laboratories. Both public and private health facilities in the region are required to send their health information data to the district and regional health administration for inclusion in the District Health Information Management System, version II (DHIMS-II), although very few private facilities comply.

Pregnancy related mortality ratio and institutional mortality ratio of the region are 378/100,000 and 169.9/ 100,000 respectively [11,12]. Skilled antenatal care coverage is 95.8%, which is comparable to the national coverage. The skilled delivery rate of between 79.0% and 84.0% is higher than the national coverage of between 55.0% and 58.0% [11,13].

### Health management information system data management process of the GHS

In Ghana, primary data capture at the facility level is paper-based using registers, forms and notebooks in the first instance. The data are subsequently collated and summarized onto nationally designed standard forms and thereafter electronically captured in the DHIMS data base at district level. This makes data accessible at the reporting health facilities and all levels of the health system simultaneously and in real time.

The primary sources of antenatal and delivery data capture at the facility level are the *Maternal Health Record Book* (which clients take home), the *Antenatal Register,* (a national register designed by the GHS), the *Expanded Programme on Immunization (EPI) tally booklet* for capturing data on Tetanus Toxoid (TT) immunization for women and the *Delivery register* (sometimes labeled *Returns on Delivery Book* or *Labour room admission and discharge book* in some facilities). Other sources of primary data capture include the facility-generated notebooks used to capture data on Hemoglobin tests done, referrals and Caesarean sections conducted, amongst others.

Data from the primary sources is summarized onto the *Midwives returns sheet* (herein described as “aggregate form”) on a monthly basis at the health facility level. The collation is mainly done by midwives. This summary is reviewed by the head of the facility before submission to the district or sub-metropolitan offices. At this point, the data are entered into the DHIMS-II database.

### Recording and compilation of data in registers at health facility level

At registration, the woman is assigned a registration number and her details are captured in the register. Variables captured in the register include the registration number, name, age, parity, hemoglobin at registration, hemoglobin at 36 weeks gestation, administration of intermittent preventive treatment in pregnancy (IPTp) doses 1, 2 & 3, TT immunizations given. Data on age, parity and hemoglobin at registration for each woman is required to be entered into the register at registration. Data on subsequent visits are to be recorded against the client’s name and registration number. For any subsequent day of antenatal visit, most of the clients doing the hemoglobin test at 36 weeks gestation (as well as those taking IPTp and TT) would have registered earlier on and so would have to have their results recorded on earlier pages of the register against their names. Collation of data at the end of the month then requires the provider to manually check the antenatal register for the client’s name using the registration number. This may imply going back many pages in order to collate the data for a particular month, depending on the number of registrants, as well as the gestational age at which the client was previously booked or registered. In some health facilities therefore, providers keep additional record books (notebooks) to record hemoglobin tests done (at registration and at 36 weeks gestation). This makes it easier for them to carry out the collation at the end of the month, though it does not allow for the linkage of hemoglobin test results to individual clients.

**Figure 1:**
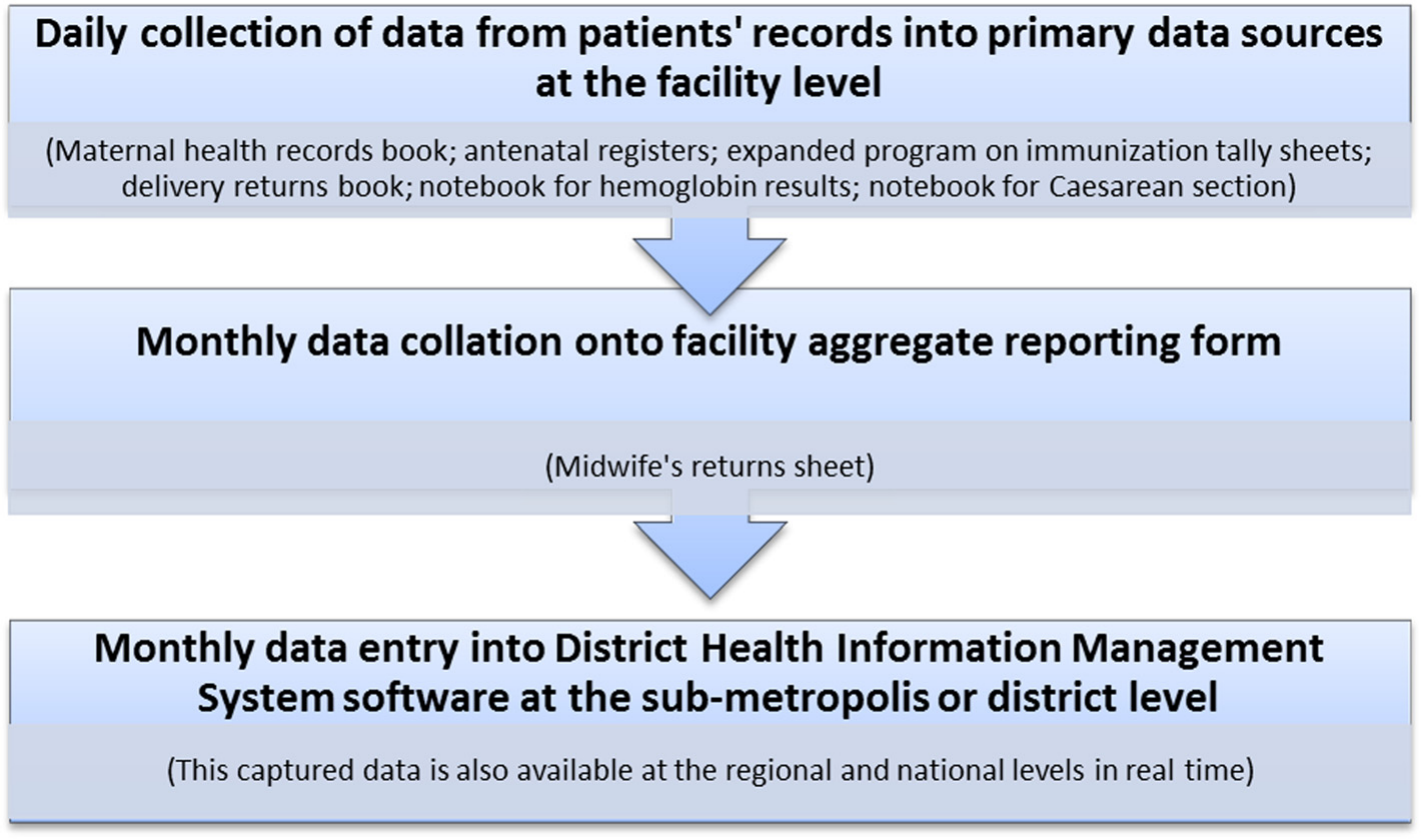
**Data management processes in the Ghana health service of the Greater Accra region of Ghana.** A description of data management processes from health facility to the regional level in the Ghana Health Service, Greater Accra region of Ghana.

All health facilities record their deliveries in a register though some facilities have a separate notebook to record deliveries by Caesarean sections. At the end of the month, the information is also summarized onto the aggregate form at the facility level before entry into the DHIMS-II. Figure 1 summarizes the maternal health service data management system as described above.

### Study design

A cross-sectional study involving review of records of maternal health service data collected during the first quarter of 2012 was conducted was conducted. Three levels of data sources were reviewed.

- Primary source data at health facility level (antenatal registers, EPI tally sheets, hemoglobin notebooks where in use, delivery registers, notebooks for caesarean sections where in use)
- Facility aggregate data (Midwives Returns sheet) and
- DHIMS-II data (district level data)

### Sampling and data collection

All districts in the region were ranked in descending order based on the total number of maternal deaths for the year 2011. The list was divided into two sets, an upper half of eight districts with the highest number of maternal deaths and a lower half of nine districts with the lowest number of deaths. Three districts were randomly selected by balloting with replacement, from each set, making a total of six districts. For each of these districts, the district hospital was selected. One other primary health facility (health center, clinic or CHPs compounds) was also randomly sampled through balloting. Thus, overall, 12 health facilities in the region had their data validated. Though private facilities were included in the sampling process, none was sampled.

For antenatal data, the variables checked for completeness and accuracy of data transfer were age, parity, number of registrants, number of attendants with hemoglobin done at registration and 36 weeks gestation, and number of attendants receiving at least the 2^nd^ dose of tetanus toxoid injection (TT2+). For delivery data, the variables assessed were total deliveries, total live births and total maternal deaths.

Using double data visual verification by two data collectors, primary source antenatal and delivery data (from antenatal registers, notebooks and delivery registers) were manually counted and recorded and used as reference. The data on the facility aggregate forms (the Midwives returns sheet) were also recorded as facility aggregate data, as well as data from the DHIMS-II for the period. The biostatistics department of the regional health service provided the DHIMS-II data for the districts involved. The three sources of data were compared to determine errors in data transfer from one source to the other. A structured checklist of variables was used to collect data on these numbers.

### Data analysis

Completeness and accuracy of data transfer from primary source to other levels were computed. The formulae used were adapted from a previous study [10].

For completeness, percentage of missing data for each of the three mandatory variables at antenatal registration (age, parity and HB at registration) was calculated

$$ \mathbf{Completeness}=\left(\mathbf{R}-{\mathbf{P}}_{\mathbf{i}}\right)/\mathbf{R}*\mathbf{100}\% $$

(Where ***R*** represents the total registrants from primary data source; ***P***_*i*_ represents number of registrants for whom data for a variable ***i*** is recorded in register.

For accuracy of data transfer, three types of percentage errors in data transfer across the three data sources were calculated [10].

$$ \begin{array}{c}\hfill \boldsymbol{Percentage}\ \boldsymbol{Error}=\left(\mathbf{Difference}\ \mathbf{between}\ \mathbf{both}\ \mathbf{data}\ \mathbf{sources}\div \mathbf{total}\ \mathbf{data}\ \mathbf{entered}\right)\times \mathbf{100}\%\hfill \\ {}\hfill =\left(\frac{{\mathrm{d}}_i-{\mathrm{d}}_{\mathrm{j}}}{\mathrm{D}}\right)\times 100\%\hfill \end{array} $$

(where ***“d “*is the actual number of data in the data source;*d*i,*d*j** represent the sequence in data gathering sources with **j** always acting as the reference for **i; and D** is the total data inspected for the variable).

*Percentage Error 1* is a measure of the error involved in the transfer of data from the primary sources to the aggregate data forms. It estimated the deviation of the facility aggregate data from the primary source data.

*Percentage Error 2* is a measure of the disparity between the data in the primary source, and what is finally received at the district and regional levels through the DHIMS-II. It estimated the deviation of DHIMS-II data from primary source data.

*Percentage Error 3* is a measure of the error that occurs during the transfer of data from the aggregate forms into the DHIMS-II software. It estimated the deviation of the facility aggregate data from the DHIMS-II data, mainly due to data entry errors.

We also computed the 95% confidence interval of the completeness and percentage error using the formula:

$$ y=p\pm Z\alpha \sqrt{\frac{p\left(1-p\right)}{D}} $$

Where ***y*** = 95% confidence interval of the estimate; ***p*** = % completeness or percentage error; ***z*** = 1,645 (1-sided alphfa of 0.05) and ***D*** = total data inspected for the variable.

SPSS version 20 was used for the analysis.

### Ethical approval

This study was carried out in partnership with the biostatistics department of the Greater Accra Regional Health Directorate. Permission was given by the directorate to use the routine secondary data from the selected facilities as well as what is available in the District Health Information Management System version II (DHIMS-II). These data are usually available to health workers with permission to use for specific purposes. No human subjects were directly interviewed or followed up. Thus no ethical approval was required.

## Results

The study was conducted in the second quarter of 2012. Though the study sample included six districts, one of the districts was unable to provide complete primary source antenatal data, which is the reference data source, because one of the antenatal primary source data registers for the period under review could not be located. Therefore the district was not included in the analysis of antenatal variables but was included for delivery variables since complete data were available for deliveries.

### Data completeness

All the available data from a total of 5,537 antenatal registrants and 3,466 deliveries seen over the period of the first quarter of 2012 and recorded into facility primary sources were reviewed. Completeness of antenatal data was 99.1% (95% CI = 98.4% – 99.8%), 98.0% (95% CI = 95.7% - 100.0%) and 85.8% (95% CI = 73.5% - 98.2%) respectively for age, parity and hemoglobin done at registration. The mean percentage completeness for these three mandatory variables at registration, from primary data source from all districts, was thus 94.3% (95% CI = 90.6% - 98.0%).

**Figure 2:**
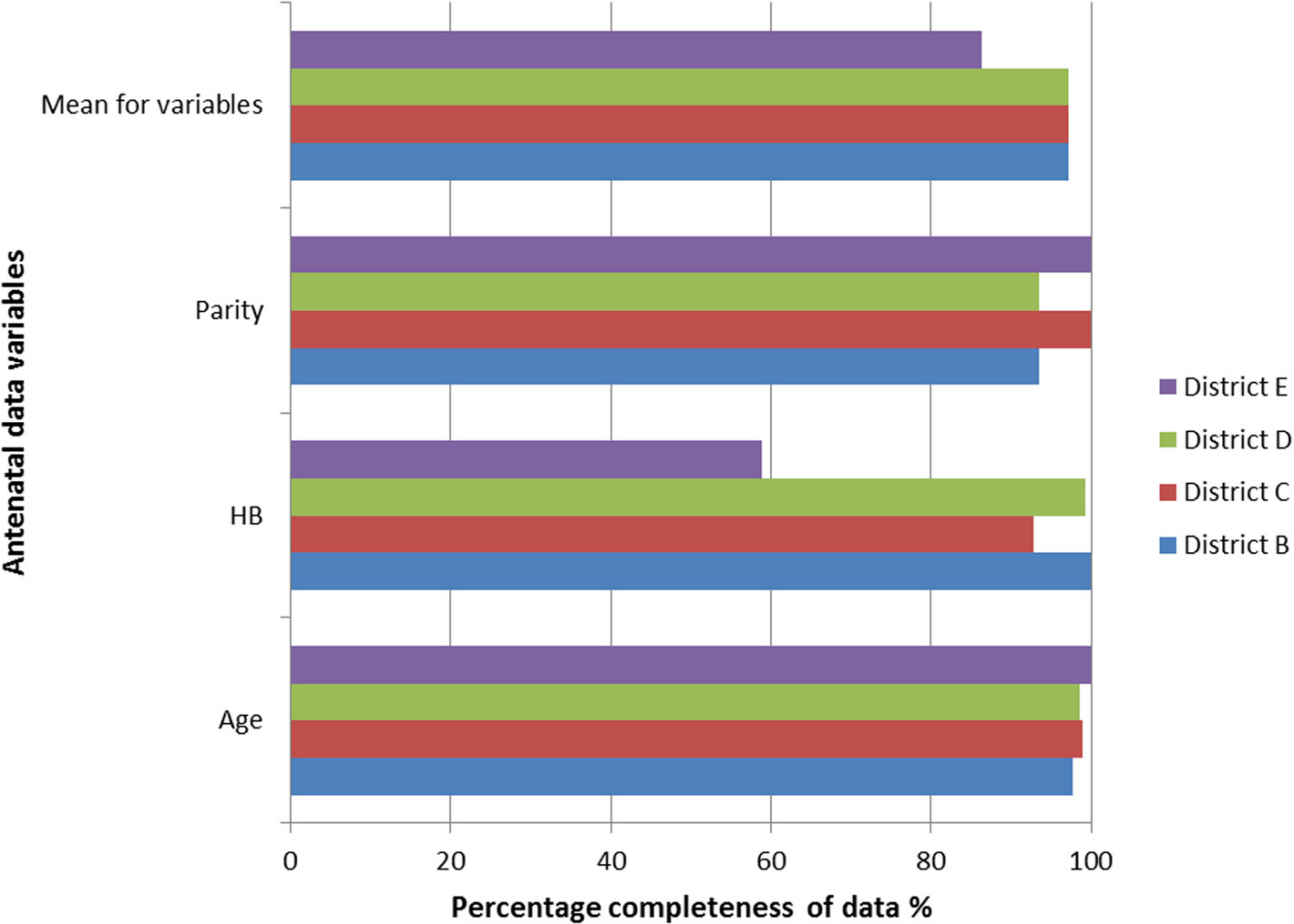
**Mean percentage completeness of primary source data in the Greater Accra region of Ghana, 2012.** A graph showing the mean percentage completeness of the primary source data for antenatal service data variables (Age, Parity and Hemoglobin at registration) by the districts sampled, in the Ghana Health Service, Greater Accra region of Ghana.

Across the 5 districts and sub-metropolises, there was some variation in the completeness of the facility primary source data, although not large. Completeness for age data ranged from 97.4% to 100.0%. The best performing district had 100.0% while the least performing district had a mean of 95.8%. Generally completeness was best for age data, followed by data on parity, and lastly hemoglobin at registration data. Mean percentage completeness for the hospitals was lower (97.3%) than that of smaller facilities such as health centers, clinics and CHPS compounds (99.6%), though this difference was not statistically significant *(p = 0.2).* Figure 2 is a graphical representation of the completeness of data for the various districts.

For the aggregate forms and DHIMS-II database, completeness was 100.0% for all the required variables on the midwives returns forms and in the database, for all facilities and districts. Details of percentage completeness by health facility and district have been provided in Table 1.

**Table 1:** **Percentage completeness of data by facility and district for three antenatal services variables (age, parity and hemoglobin at registration) in the Ghana Health Service, Greater Accra region, Ghana**

**Facility/(District)**	**Registrants (a)**	**Number of registrants with age recorded (b)**	% completeness for Age (X) = (b/a*100%)	**Number of registrants with parity recorded (c)**	% completeness for parity (Y) = (c/a*100%)	**Number of registrants with hemoglobin at registration recorded (d)**	% completeness hemoglobin at registration (Z) = (d/a*100%)	**Mean % completeness (X+Y+Z)/3**
1	809	809	100.0	742	91.7	770	95.2	95.6**
2	56	56	100.0	56	100.0	56	100.0	100.0
(A)	865	865	100.0	798	92.3	826	95.5	95.9
3	911	891	97.8	848	93.1	910	99.89	96.9**
4	66	64	97.0	66	100.0	66	100.00	99.0
(B)	977	955	97.8	914	93.6	976	99.9	97.1
5	166	165	99.4	166	100.0	116	69.9	89.8
6	533	526	98.7	533	100.0	533	100.0	99.6**
(C)	699	691	98.9	699	100.0	649	92.9	97.2
7	1406	1384	98.4	1313	93.4	1406	100.0	97.3**
8	41	41	100.0	41	100.0	31	75.6	91.9
(D)	1447	1425	98.5	1354	93.6	1437	99.3	97.1
9	1390	1390	100.0	1388	99.9	765	55.0	85.0**
10	159	157	98.7	159	100.0	148	93.1	97.3

District totals **Figures are for bigger facilities (Hospitals).

### Accuracy of data transfer

**Figure 3:**
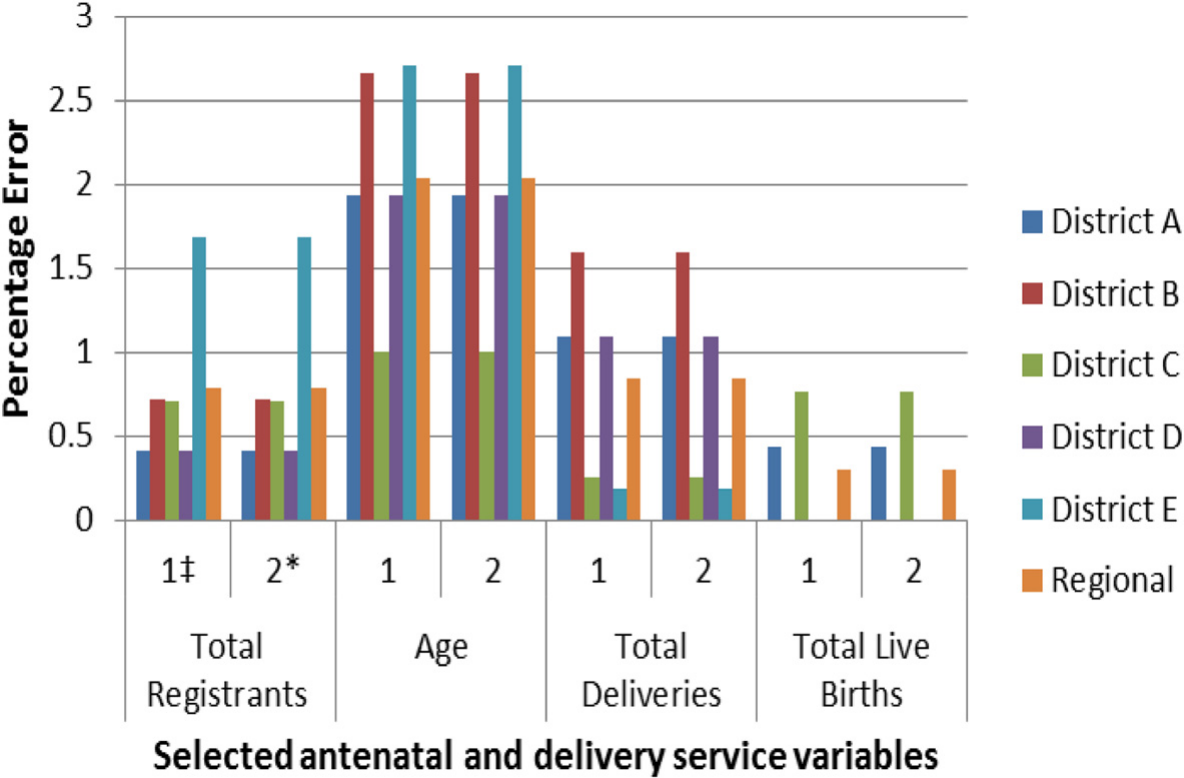
**Percentage error in data transfer of selected antenatal and delivery service variables in the Greater Accra region of Ghana, 2012.** A graph showing the percentage error in data transfer for some selected antennal and delivery service variables (total registrants, age, total deliveries and total live births) by the districts sampled in the Ghana Health Service, Greater Accra region of Ghana. ‡*Error 1* is a measure of the disparity between the data in the primary sources and that in the aggregate data forms. *Error 2 is a measure of the disparity between the data in the primary source, and that in the web-based DHIMS-II.

Three sources of data were compared in this study. The most consistent variable in terms of accuracy of data transfer was *maternal death*, with a percentage error of 0.0% across all facilities, districts and sub-metropolises. Percentage errors 1 and 2 were similar for the variables *age, parity, total number of women with hemoglobin done at registration, total number of women receiving TT2+ and total number of registrants.* For these variables the two percentage errors ranged from 0.0% to 4.9%. Percentage error 3 was generally very low or 0.0%, which means that there is very little or no error in transferring the facility aggregate data to the DHIMS-II. Generally, percentage errors 1 and 2 for the variable *total number of women with hemoglobin checked at 36 weeks gestation* were high, ranging from 14.1% to 35.6%. The overall percentage error in transfer of the data sampled was 1.0% (95% CI = 0.8% - 1.2%). Figure 3 summarizes the percentage errors for some of the variables across the district while percentage errors in data transfer for all the districts in our sample for antenatal and delivery data are shown in tables in the Tables 2 and 3 respectively.

**Table 2:** **Percentage errors in transfer of antenatal service data for total regional sample in the Ghana Health Service, Greater Accra region, Ghana**

**HEALTH INDICATOR**	**PRIMARY SOURCE DATA (as counted)**	**FACILITY AGGREGATE DATA (a)**	**DHIMS -II DATA**	**TOTAL DATA ENTERED (a + 2)**	**PERCENTAGE ERROR-1 (%)**	**PERCENTAGE ERROR-2 (%)**	**PERCENTAGE ERROR-3 (%)**
Total registrants	5537	5557	5557	5539	0.4	0.4	0
Age	5490	5556	5555	5539	1.2	1.2	0.02
Total number of women receiving TT2+	3655	4155	4155	3657	13.7	13.7	0
Total number of women with hemoglobin checked at registration	4802	5057	5057	5539	4.6	4.6	0
Total number of women with hemoglobin checked at 36 weeks gestation	1494	2027	2238	1496	35.6	49.7	14.1
Parity	5306	5577	5576	5539	4.9	4.9	0.02

*Error 1* is a measure of the disparity between the data in the primary sources and that in the aggregate data forms.

*Error 2* is a measure of the disparity between the data in the primary sources and that in the web-based DHIMS-II.

*Error 3* is a measure of the disparity between the facility aggregate data and that in the web-based the DHIMS-II.

**Table 3:** **Percentage errors of delivery service data transfer for total regional sample in the Ghana Health Service, Greater Accra region, Ghanak**

**HEALTH INDICATOR**	**PRIMARY SOURCE DATA (as counted)**	**FACILITY AGGREGATE DATA (a)**	**DHIMS – 2 DATA**	**TOTAL DATA ENTERED = (a + 2)**	**PERCENTAGE ERROR-1 (%)**	**PERCENTAGE ERROR -2 (%)**	**PERCENTAGE ERROR-3 (%)**
Total deliveries	3466	3483	3531	3465	0.5	1.9	1.4
Total live births	3411	3456	3497	3468	1.9	1.8	0.1
Total maternal deaths	0	0	0	2	0	0	0

*Error 1* is a measure of the disparity between the data in the primary source and that in the aggregate data forms.

*Error 2* is a measure of the disparity between the data in the primary source and that in the web-based DHIMS-II.

*Error 3* is a measure of the disparity between the facility aggregate data and that in the web-based the DHIMS-II.

## Discussion

### Results in context

The results of this study show that, overall, there are small variations in the completeness and accuracy of data transfer of primary source data at the facility and district levels, and this should be carefully considered when using the data. Underlying these variations is the fact that recording of data into these sources is largely manual and paper – based. However, once the data are captured in the aggregate forms, they are accurately transferred on to the next level. At the time of conducting this study, the GHS’s Centre for Health Information Systems (CHIM) had introduced data quality audits in 2011 but had not yet developed Standard Operating Procedures (SOPs) for data management, nor defined the level of accuracy and completeness that should be achieved. It was therefore unclear as to what acceptable standards of data quality are for Ghana. Although there are no global and uniformly defined guidelines on the subject, the quantification of data quality using percentage error from the sources of data is acceptable, as done in individual studies, and is the basis of the percentage errors calculated in this study [10]. In an assessment of primary care health data in Mozambique, completeness of manual data was between 37.5% and 52.1% [14]. One study also established a mean percentage completeness from 94.0% to 97.0% using information system data to assess maternal and perinatal care services completeness [15]. Our study findings of 94.3% completeness for the selected antenatal variables, although similar to these studies, can be further improved, especially with regards to data on hemoglobin done at registration. Omission of important maternal health data in registers in Ghana has been identified in an earlier study in Kumasi in the Ashanti region [5]. This includes information on age and parity amongst others. For hemoglobin test at registration, it often depends on whether the facility has a laboratory or not. Where clients have to do their laboratory tests outside the facility, sometimes they do not return with the results the same day for it to be recorded. A few clients may also not have the tests requested for them for various reasons, or do not do the requested tests at all. These reasons may account for why some clients do not have the data on the three variables recorded, as well as omission on the part of the provider, either from failure to ask or to record the given answer in the register.

The visual data verification method of data quality inspection which was employed in this study, allows for error rate of transfer of data from primary source to next level (aggregate form or electronic database) of a maximum of 3.3% [10]. In a study in Mozambique, differences between manually recorded and electronic data sources were computed and the majority (86%) of the four sites used had differences between data sources of 10.0% or less [14]. Comparison of manual medical records of confirmed cases of diabetes from primary source data with electronic data yielded accuracy of 96.7%, with a discrepancy of about 4.0% in another study [16]. Our estimates of percentage error in data transfer of 0.0% -5.0% range and average of 1.0% are consistent with percentage errors detected in some large clinical databases [17], except for *total number of women receiving TT2+* and *total number of women with hemoglobin checked at 36 weeks gestation, which were higher.* This indicates that the aggregate data is comparable to primary source data.

### Addressing challenges

Hemoglobin measurements at registration and at 36 weeks gestation are mandatory for women attending antenatal clinic in Ghana. Percentage Errors 1, 2 and 3 for *total number of women with hemoglobin done at 36 weeks gestation* are rather high. Potential sources of error exist depending on who is entering or collating the data, whether there is availability of alternate books for keeping this record or not as well as the volume of data, amongst others. What was not clear is the reason why facilities without a laboratory were able to organize for hemoglobin tests done at registration but not at 36 weeks gestation. This could be further explored. For the district that recorded the lowest percentage error 1 for this variable, this might be attributed to the use of another notebook to record this data, making counting easier, which was observed. This approach of recording data however has the disadvantage of not allowing one to link the results to patient individual data. It also made it difficult to assess whether a particular patient’s hemoglobin had improved since registration or not. A thorough investigation of this finding on HB data would be useful, since hemoglobin at 36 weeks gestation remains a useful indicator as to how well the pregnant women are prior to going into labor. This is especially critical in an environment where postpartum hemorrhage remains a major cause of maternal morbidity and mortality [18-20].

Previous studies have shown that in some health systems the culture of using information for decision making is not well developed [4,21-23]. However, even with sophisticated electronic data collection and collation systems in place, clinical staff need to be equipped with the skills of data analysis, interpretation and use in their routine work [24,25]. Also, they must collect data that is linked to indicators for which there are clear actionable responses [24]. These measures will ensure optimal utilization of output from the health information management system. A synthesis of this process must exist not only at the national, regional and district levels, but also at the facility level, so that the facility can generate its own important dataset that serves as a guide to influence local decision making on quality of care.

One key issue is the lack of an internal quality process in the health information system that ensures that data is validated regularly at all levels. This problem has also been reported in other countries through data validation studies [26]. The forms and registers available for data capture and transfer should have checks incorporated into them to minimize errors. Better still these forms can be made electronic with checks incorporated into them. Adequate training and supervision for data handlers is required to ensure that they generate and accurately transfer complete data. Detection of errors should not be left to the ingenuity of data handlers. Where performance indicators are related to figures being reported, the lack of such internal quality control mechanisms will potentially encourage under-reporting of bad outcomes and over-reporting of good outcomes. The consistent use of the recently developed and printed Standards Operating Procedures (SOPs) for data management in the GHS should be greatly encouraged.

The manual generation of daily service delivery data is another challenge that needs to be addressed. Manual data collection of data overburdens health facility staff and is prone to errors [7]. It can potentially affect the output of the staff in terms of service delivery. Electronic systems are often more efficient as they often lend themselves to more rigorous internal checks [27]. In an environment where health workers and especially midwives are inadequate, the few are overburdened with data management. Use of dedicated workers for data management will not only free the midwives and other clinical staffs some time for their work, but also improve the quality of data generated [24].

### Study limitation

Due to the fact that no private facility was sampled in our study, the results here are applicable to only public sector health facility data. Also, the high percentage error in transfer of data on hemoglobin done at 36 weeks gestation is worrying but the scope of our study did not allow us to explore the factors responsible for these results. We recommend other studies to look into those issues. Finally, we used the primary source data as reference for comparing the other sources of data. Any shortcoming in capturing data into these primary sources will therefore reflect on our results.

## Conclusion

The routine maternal health service data in the Greater Accra region, available through the DHIMS-II database is relatively complete and accurately reflects what exists in primary facility level sources. This makes it an effective tool for monitoring the MGDs 4 and 5, as well as guide the implementation of future interventions and research.
